# Attenuation of *Clostridioides difficile* Infection by *Clostridium hylemonae*

**DOI:** 10.4014/jmb.2510.10017

**Published:** 2026-01-13

**Authors:** Sueun Choi, Heewon Kwon, Woon-Ki Kim, GwangPyo Ko

**Affiliations:** 1 Department of Environmental Health Sciences, Graduate School of Public Health, Seoul National University, Seoul 08826, Republic of Korea; 2 Brain Korea for Global Leader Of Better Environmental health (BK4GLOBE), Republic of Korea; 3 Institute of Health and Environment, Seoul National University, Seoul 08826, Republic of Korea

**Keywords:** *Clostridioides difficile* infection (CDI), *C. hylemonae* DSM 15053, Gut microbiota, 7α-Dehydroxylation, Secondary bile acids, Fecal microbiota transplantation, Gut microbiota, Fecal

## Abstract

*Clostridioides difficile* infection (CDI) is a bacterial infection of the colon that can cause diarrhea and colitis. The use of antimicrobials disrupts the intestinal microbiota, weakening colonization resistance and creating an environment in which *C. difficile* can establish infection. It is, therefore, necessary to identify specific bacteria that are helpful for the recovery of the intestinal microbiota in individuals with CDI. Previous studies have identified several strains that showed a negative correlation with *C. difficile*. Among these strains, *C. hylemonae* DSM 15053, which possesses the *bai* operon similar to *Clostridium scindens*, was selected. To test this hypothesis, we utilized a CDI mouse model and evaluated the inhibitory effect of *C. hylemonae* DSM 15053. Furthermore, to gain insights into the underlying mechanisms, we performed gut microbiota analysis. Contrary to our expectations, *C. hylemonae* DSM 15053 did not significantly produce SBAs. Interestingly, however, microbial diversity and richness were significantly higher in the *C. hylemonae* DSM 15053–treated group compared with the PBS control group. In addition, we observed a higher abundance of the genera *Phocaeicola*, *Akkermansia*, and *Parabacteroides* in the *C. hylemonae* DSM 15053 group. Moreover, metagenomic and metabolomic analyses revealed that *C. hylemonae* DSM 15053 mitigates CDI through a mechanism distinct from that of *C. scindens* KCTC 5591, which primarily functions as a regulator of bile acid metabolism.

## Introduction

*C. difficile* is a spore-forming, Gram-positive anaerobe recognized as the leading cause of antibiotic-associated colitis and healthcare-associated infectious diarrhea [1-3]. It manifests in a spectrum of clinical outcomes, ranging from asymptomatic colonization to fulminant colitis and toxic megacolon. Using surveillance data from the CDC EIP across 10 U.S. sites, the estimated national burden of CDI declined from 2011 through 2017 (476,400 cases; 95% confidence interval [CI], 419,900–532,900 in 2011; 462,100 cases; 95% CI, 428,600–495,600 in 2017). In contrast, a study using the Korean National Health Insurance Service-National Sample Cohort (NHIS-NSC) reported a steady increase in CDI incidence, from 0.030% in 2006 to 0.317% in 2015 [4]. These epidemiological trends emphasize the urgent need to elucidate CDI pathogenesis and develop targeted therapeutic strategies.

Several factors contribute to CDI, including genetics, diet, and age [5]. Extended or repeated exposure to antibiotics during hospitalization profoundly perturbs the normal gut microbiota, which is widely recognized as a primary predisposing factor for CDI. The onset of CDI and its clinical manifestations stem from antibiotic-induced dysbiosis [6]. In individuals treated with antibiotics, dormant *C. difficile* spores can germinate, expand as vegetative cells, and release key exotoxins—TcdA and TcdB—that drive the pathogenicity of *C. difficile* [7].

Secondary bile acids (SBAs), derived from primary bile acids (PBAs) through microbial metabolism, are key mediators in the gut [8]. SBAs play important roles in modulating *C. difficile* spore germination and growth, often in cooperation with the gut microbiota. Among SBAs, deoxycholic acid (DCA) and lithocholic acid (LCA) are known to inhibit *C. difficile* growth [9]. However, only a limited number of bacteria from the Lachnospiraceae and Ruminococcaceae families possess the 7α-dehydroxylation capability to convert cholic acid (CA) and chenodeoxycholic acid (CDCA) into DCA and LCA, the two most common SBAs [10, 11]. Thus, understanding SBAs production by commensal bacteria within the gut microbiota is essential for reducing the incidence of CDI.

The intestinal microbiota plays a crucial role in suppressing *C. difficile* by maintaining microbial diversity and metabolic balance. Antibiotic-induced dysbiosis reduces short-chain fatty acid (SCFA) production, disrupts bile acid metabolism, and weakens mucosal defenses, thereby facilitating *C. difficile* germination and growth. Clinical studies have shown that reduced microbial diversity is associated with more severe and recurrent CDI. The presence of key bacterial groups such as Firmicutes and Bacteroidetes is linked to colonization resistance. However, the mechanisms underlying these protective effects remain incompletely understood.

In previous studies using LDA effect size (LEfSe) analysis, several bacterial taxa negatively correlated with *C. difficile* were identified [12]. Among them, *C. hylemonae* DSM 15053, a bile acid 7α-dehydroxylating intestinal bacterium possessing the bai operon gene cluster responsible for SBAs biosynthesis, was selected for further study [13, 14]. As noted above, SBAs play a crucial role in controlling *C. difficile* spore germination and growth. *C. scindens* KCTC 5591 is also a well-known bile acid 7α-dehydroxylating bacterium. Indeed, a previous study by Buffie *et al*. demonstrated that *C. scindens* inhibits CDI by converting PBAs into SBAs. Based on this evidence, we hypothesized that *C. hylemonae* DSM 15053 might also exert inhibitory effects on CDI.

To test this hypothesis, we utilized a CDI mouse model to evaluate the inhibitory effects of *C. hylemonae* DSM 15053. Furthermore, to elucidate the underlying mechanisms, we performed gut microbiome profiling to determine whether there were differences in specific bacterial taxa between groups, as well as metabolomic analyses to assess the ability of *C. hylemonae* DSM 15053 to produce SBAs.

## Materials and Methods

### *In Vivo* CDI Experiments Using Conventional Mouse

C57BL/6N mice (6-7 weeks old, female, 4 mice/cage) were purchased from Orient Bio (Republic of Korea) and maintained in ABL-2 (animal biosafety level 2) facility at Seoul National University. Conventional mice experiments were conducted at Seoul National University Animal Center for Pharmaceutical Research. Conventional mice experiments were conducted using C57BL/6N, 6-7 weeks old mice.

All *in vivo* experiment were performed after 1 week of stabilization period. Before *C. difficile* spore challenge, 7-8 weeks old mice were treated with antibiotics cocktail containing kanamycin (0.4 mg/ml), gentamicin (0.035 mg/ml), colistin (850 U/ml), metronidazole, (0.215 mg/ml) and vancomycin (0.045 mg/ml). Clindamycin (20 mg/kg) was injected intraperitoneally 24 h before infection. Mice were inoculated with 1 * 10^4^ cells *C. difficile* spores in 200 μl of PBS via oral gavage. A stainless steel fastening plate was used to prevent coprophagy in mice. All animal experiments were conducted under as approved by the Institutional Animal Care and Use Committee (IACUC) of Seoul National University. The permit number of conventional mice experiments are SNU-200709-1-3.

### *C. scindens* KCTC 5591 and *C. hylemonae* DSM 15053 Culture Condition

*C. scindens* KCTC 5591 was cultured in gifu anaerobic medium (GAM) broth media and *C. hylemonae* DSM 15053 was cultured in gifu anaerobic medium (GAM) broth media supplemented with CA 1mM (w/v), CDCA 1 mM (w/v). All bacteria were cultured at 37°C for 24 h under anaerobic condition with anaerobic chamber and anaerobic jar.

### *C. difficile* Culture Conditions

*C. difficile* *ATCC 43255* (VPI 10463) was obtained from the American Type Culture Collection (ATCC, USA). The strain was grown anaerobically at 37°C for 24 h in brain heart infusion medium supplemented with 0.5% yeast extract, 0.1% cellobiose, 0.1% maltose, and 0.05% L-cysteine (BHIS). Anaerobic conditions were maintained using an AnaeroPack rectangular jar with AnaeroPack-Anaero gas-generating sachets (Mitsubishi Gas Chemical, Japan).

### Sporulation of *C. difficile*

*C. difficile* ATCC 43255 (VPI 10463) was streaked onto SMC agar (per liter: 90 g Bacto peptone, 5 g protease peptone, 1 g NH_4_SO_4_, 1.5 g Tris base, and 15 g agar) and incubated anaerobically at 37°C for 7 days to induce sporulation. The bacterial growth was then harvested into 1 ml of sterile PBS and centrifuged at 13,000 rpm for 10 min. The pellet was washed once with deionized distilled water and centrifuged again at 13,000 rpm for 5 min. After discarding the supernatant, the sample was treated with 100% ethanol and incubated at 70°C for 1 h to eliminate vegetative cells. The suspension was subsequently centrifuged at 13,000 rpm for 5 min, resuspended in DIW, and stored at −80°C until further use [15].

*C. difficile* ATCC 43255 (VPI 10463) was inoculated on SMC agar (90 g Bacto peptone, 5 g protease peptone, 1 g NH4SO4, 1.5 g Tris base, and 15 g agar per liter) and cultured anaerobically at 37°C for 7 days. The cultured *C. difficile* ATCC 43255 (VPI 10436) was suspended in 1 ml sterilized PBS and centrifuged at 13,000 rpm for 10 min. After discarding the supernatant, it was resuspended in deionized distilled water (DIW) and centrifuged at 13,000 rpm for 5 min. After removal of the supernatant, treated with 100% ethanol and left at 70°C for 1 h to kill vegetative cells. Finally, after centrifugation at 1300 0rpm for 5 min, it was suspended in DIW and stored at -80°C until use [15].

### Measurement of Clinical Score

Throughout the post-infection period, clinical symptoms were monitored daily for each mouse. The final clinical score was calculated as the sum of all individual parameters and ranged from 0 (normal) to 15. Mice that became moribund were euthanized, and those that died were assigned a maximum score of 15 [16].

### Measurement of *C. difficile* Colony Forming Unit (CFU) and Toxin in Mouse Stool

For quantification of *C. difficile* viable cells and spores, 1:10 serial dilutions of weighted stool samples suspended in PBS in anaerobic chamber were plated on ChromID *C. difficile* agar (bioMérieux, France) and incubated at 37°C for 24 h under anaerobic condition [17]. C.difficile toxin A and B titer *in vivo* samples were quantified by *C. difficile* TOX A/B II (TECHLAB, USA) [18].

### Polymerase Chain Reaction (PCR) Assay of baiCD

PCR amplification of the *baiCD* gene was performed using genomic DNA from bile acid 7α-dehydroxylating bacteria. Each reaction (50 μl) contained 200 ng template DNA, 0.5 μmol/L of each primer, and 0.6 U of Platinum Pfx DNA polymerase (Invitrogen, USA). The reaction mixture consisted of 1× Pfx amplification buffer, 1.5 mmol/L MgSO_4_, 5% DMSO, 10 μg BSA, and 950 μmol/L dNTPs (312.5 μmol/L of each nucleotide), with sterile ultrapure water added to the final volume. PCR cycling conditions were: initial denaturation at 94°C for 2 min; 35 cycles of 94°C for 20 sec, 52°C for 30 sec, and 69°C for 90 sec; followed by a final extension at 68°C for 10 min. Amplified products were resolved on 1% SeaKem agarose gels in a Tris–acetate–EDTA buffer, stained with ethidium bromide, and visualized under UV illumination. Gel images were acquired using a Fotodyne Foto/Analyst Investigator system (USA) and analyzed with NIH Image 1.62.

### Quantification of Concentration of SBAs *in vitro* and Metabolomics

Bacteria culture 1 ml was used to quantify SBAs. To extract bile acids and other metabolites, an equal volume of 80% methanol was added to the sample. The mixture was sonicated for 3 min, centrifuged at 13,000 rpm for 1 min, and the resulting supernatant was filtered through a 0.22-μm membrane. The filtrate was evaporated using a SpeedVac concentrator (Germany), yielding a dried metabolite residue containing bile acids, which was stored at −80°C until analysis. Separation of bile acids—including CA, CDCA, LCA, DCA, TCA, TDCA, TUDCA, and UDCA—was performed using an Acquity UPLC BEH C18 column (100 mm × 2.1 mm, 1.7 μm) on an Acquity UPLC system (Waters). Chromatographic conditions were as follows: mobile phase A, water with 0.1% formic acid; mobile phase B, acetonitrile with 0.1% formic acid; injection volume, 2 μl. The mobile-phase B gradient was increased from 5% to 90% over 15 min at a flow rate of 0.4 mL/min. Qualitative analysis of bile acids was carried out using a Waters Synapt G2-Si Q-TOF mass spectrometer equipped with an electrospray ionization (ESI) source operating in negative ion mode with tofMRM acquisition. Mass spectrometer settings for the Synapt G2-Si included: capillary voltage, 25 kV; source temperature, 100°C; sampling cone, 40; source offset, 80; desolvation temperature, 250°C; cone gas, 50 L/h; desolvation gas, 600 L/h; and nebulizer gas, 6.5 bar. Bile acid quantification was performed using the QuanLynx module of the MassLynx software suite [19].

Metabolite profiling of culture supernatants was conducted under positive ionization mode using MSe scanning with the same chromatographic column and UPLC conditions described above. The mass detection range was set from 50 to 1200 Da with a scan time of 0.2 s. The mass spectrometer parameters included: capillary voltage, 2 kV; source temperature, 120°C; sampling cone, 40; source offset, 80; desolvation temperature, 400°C; cone gas, 50 L/h; desolvation gas, 600 L/h; and nebulizer gas, 6.5 bar. Instrument control and data acquisition were performed using MassLynx 4.1 (Waters) [20].

For intergroup metabolic analysis, raw MassLynx data were imported into Progenesis QI (Waters). Alignment parameters were set to a retention-time window of 0.20 min and a mass tolerance of 1.0 ppm. Subsequently, metabolites were filtered using ANOVA p-values and maximum fold change criteria before exporting the dataset to EZinfo software (version 3.0.3.0) for principal component analysis (PCA) [21].

For histopathological examination, mice were euthanized on day 7 post-infection, and lung tissues were collected, fixed in 10% neutral-buffered formalin, embedded in paraffin, sectioned at 4-μm thickness, and stained with hematoxylin and eosin (H&E). Slides were digitally scanned by T&P BIO (Republic of Korea), and micrographs (10× magnification) were acquired using a Panoramic SCAN II digital slide scanner (3DHistech Ltd., Hungary).

### DNA Extraction and 16S rRNA Sequencing

Total genomic DNA from samples were extracted with QIAamp Fast DNA Stool Mini Kit (Qiagen #51604) as recommended by the manufacturer. From 515F to 926R region (V4-V5 hypervariable region) was selected in 16S rRNA gene, and amplified with the universal primers for the 515F and 926R. Then, amplicons were purified by QIAquick PCR Purification Kit (Qiagen #28104) and sequenced by a Illumina MiSeq platform (Illumina, USA) [22]. Raw MiSeq sequencing data were implemented by Quantitative Insights into Microbial Ecology2 (QIIME2) software (ver. 2024.10) [23] with Greengenes ver. 2022.10 database [24]. We firstly utilized Divisive Amplicon Denoising Algorithm 2 (DADA2) for denoising sequences, and representative sequences were collected to OTUs having 97% of nucleotide identity. Singletons and rare OTUs were excluded and relative abundances of microbial taxa were achieved using a table of non-rarefied OTUs [25, 26].

### Bioinformatic Analyses for Fecal Microbiota

All analyses were done under environments of R language (version 4.4.2) and R studio (version 2024.12.1+563), and using Ubuntu LTS 24.04.1 on Windows Subsystem for Linux. Alpha diversities were suggested measuring observed OTUs and Chao1 richness. For beta diversity analysis, PCoA analysis was conducted to evaluate dissimilarities between samples. Data visualization for the analytical results was accomplished through the following packages: phyloseq [27], ggplot2 [28], vegan [29], gridExtra [30], dplyr [31], plotly [32], tidyverse [33].

Taxonomic analysis was performed with phyloseq object by phyloseq package. Percentage relative abundance at the phylum level was calculated by the dplyr [34]. package and visualized by ggplot2. The network parameters were analyzed and graphically represented. Following R packages, ggplot2, compositions, network [35], igraph [36] were used for the microbiome co-occurrence network analysis. Marker taxa were identified using LEfSe analysis [37] implemented in the ‘microbiomeMarker’ R package.

Functional predictions were generated using PICRUSt2 (v2.4.1), with annotations based on the Kyoto Encyclopedia of Genes and Genomes (KEGG) orthologous gene family database (Kanehisa Laboratories, Kyoto University) [38]. Pearson correlation analysis between the PICRUSt2-predicted functional profiles and experimentally measured *C. difficile* gene copy numbers was performed in Python using publicly available libraries, including pandas, numpy, scikit-learn, matplotlib, and seaborn.

### Statistical Analysis

Experimental results were analyzed for statistical significance using GraphPad Prism v10 (GraphPad Software Inc.). Statistical information for each experiment is specified in the figure legends. A non-parametric Mann-Whitney U test was performed to compare the significant differences between the two groups. P values of less than 0.05 were considered as statistically significant and indicated in the figures (*P < 0.05, **P < 0.01, ***P < 0.001, ****p < 0.0001). All the statistical tests used were two-sided.

## Results

### *C. hylemonae* DSM 15053 Attenuates Clinical Symptoms of CDI

**Fig. 1:**
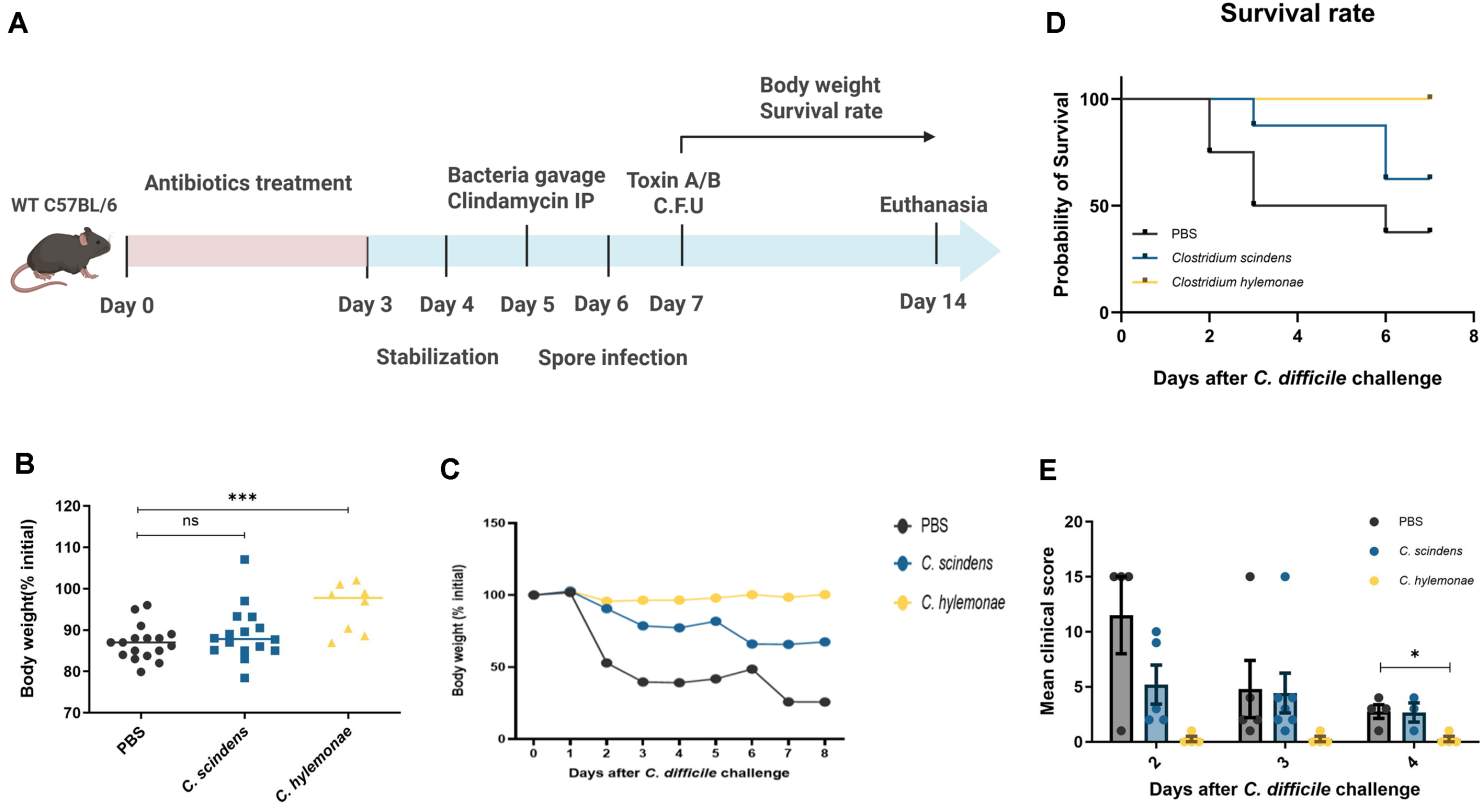
CDI mouse model and Mice body weight change after CDI and survival change, clinical score. CDI mouse model for C57BL/6 mice (**A**), The difference in body weight between groups at 2 days after CDI (**B**), The change in body weight of the whole group for 8 days after CDI (**C**), Mortality for 8 days after CDI (**D**), The clinical score of the infectious mice for 4 days after CDI (**E**). (n = 4 to 16 per group), The Mann–Whitney test was used for comparisons of continuous variables between two groups with similar variances. * : *P* < 0.05, ** : 0.01, *** *P* < 0.001, **** *P* < 0.0001. Error bars are ±SEM.

After CDI induction, changes in symptoms between groups were monitored to evaluate the effects of CDI-inhibitory strains. Key phenotypes, including body weight loss, mortality, and clinical scores, were recorded daily for eight days post-infection (Fig. 1A). No significant change in body weight was observed within the first 24 h after infection; however, the weight of all groups decreased rapidly from 24 to 48 h (Fig. 1B and 1C). Among the three groups, the *C. hylemonae* DSM 15053 group lost less than 7% of their body weight, whereas the PBS group showed a 15% loss and the *C. scindens* KCTC 5591 group exhibited an 11% loss.

The mortality rate showed a pattern consistent with body weight changes. The survival rate of the *C. hylemonae* DSM 15053 group was 87.5%, compared with 68.7% for the *C. scindens* KCTC 5591 group and 50% for the PBS group (Fig. 1D). Significant differences were also observed in clinical scores. Following infection, the PBS group exhibited the most severe symptoms during days 1–4 post-infection, resulting in higher clinical scores compared with the other groups (Fig. 1E). Mice in the *C. hylemonae* DSM 15053 group showed only mild diarrhea and ungroomed hair, whereas mice in the PBS and *C. scindens* KCTC 5591 groups displayed rapid progression of diarrhea, hunched posture, and ungroomed hair, which contributed to elevated clinical scores relative to the *C. hylemonae* DSM 15053 group.

### *C. hylemonae* DSM 15053 Inhibit Growth, Toxin Expression of *C. difficile*

**Fig. 2:**
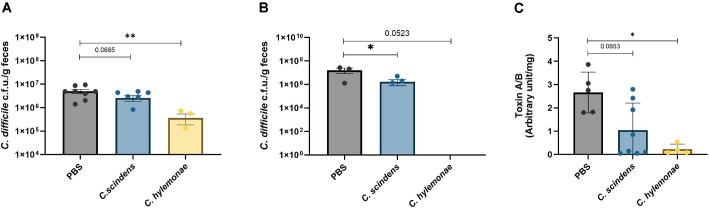
Measurement of *C. difficile* CFU and Toxin A&B. Measured *C. difficile* (CFU/g) in the feces of 1 day after CDI and 8 days after CDI (**A, B**). Toxin A & B measurement in feces of 1 day after CDI (**C**). (n = 4 to 8 per group) The Mann–Whitney test was used for comparisons of continuous variables between two groups with similar variances. * : *P* < 0.05, ** : 0.01, *** *P* < 0.001, **** *P* < 0.0001. Error bars are ±SEM.

Fecal samples collected 24 h post-infection were used to measure the abundance of *C. difficile* CFU and toxin A&B titers. Consistent with the

patterns observed in weight loss and mortality, we found significant statistical differences in toxin A/B titers among the groups. A significant reduction in *C. difficile* CFU. was observed at 24 h post-infection in the *C. hylemonae* DSM 15053 group compared with the PBS group (Fig. 2A). Complete eradication of *C. difficile* CFU is essential to reduce the risk of recurrence in CDI. To evaluate bacterial clearance, we measured *C. difficile* CFU at 8 days post-infection. Substantial clearance was observed in the *C. hylemonae* DSM 15053–treated group, whereas only a modest reduction was noted in the *C. scindens* KCTC 5591 group (Fig. 2B). A similar trend was also observed in toxin A&B titers (Fig. 2C).

### Confirmation of baiCD Gene and Enzymatic Activity of *C. hylemonae* DSM 15053 via Gut Metabolomic Analysis

**Fig. 3:**
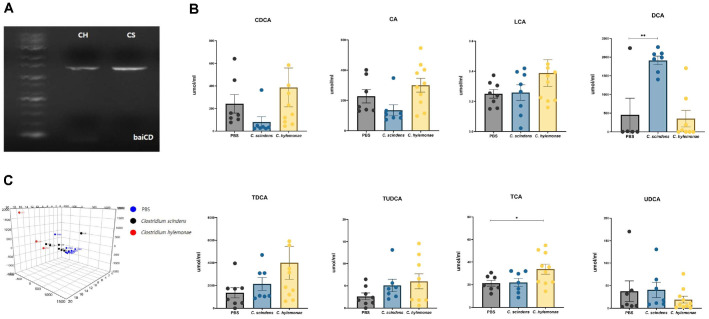
PCR result with baiCD specific primer, Metabolomic Analysis. All tested intestinal isolates were positive. PCR products were sequenced and identified as baiCD region via BLASTn, Identification of Enzyme activity of baiCD gene (**A**), Quantification of PBAs and SBAs in the *C. hylemonae* DSM 15053 culture in yBHI broth with 0.01% CA, LCA (**B**), Gut metabolome analysis via Progenesis QI at 2 days after the CDI Results of inter-group PCA input with statistically significant 11,088 metabolites among 26,272 total metabolites (**C**) (n = 3 to 10 per group), CS, *C. scindens*, CH, *C. hylemonae*. The Mann–Whitney test was used for comparisons of continuous variables between two groups with similar variances. * : *P* < 0.05, ** : 0.01, *** *P* < 0.001, **** *P* < 0.0001. Error bars are ±SEM.

There are several pathways involved in bile acid metabolism in the host. Among them, 7α-dehydroxylation has been highlighted in several studies as a factor associated with CDI. The responsible enzymes are encoded in the *bai* operon. Many studies have reported that most *Clostridium* species harbor the *bai* operon, including *C. scindens* KCTC 5591. In particular, *C. scindens* KCTC 5591 is well known for possessing the *bai* operon, and numerous studies have revealed that this strain exerts inhibitory effects on CDI. In this study, to determine whether our tested strain, *C. hylemonae* DSM 15053, carries the *bai* operon, we performed a PCR assay using *baiCD* primers. As expected, *C. hylemonae* DSM 15053 was positive for *baiCD*, similar to *C. scindens* KCTC 5591 (Fig. 3A).

Next, we quantified the bile acid concentrations in *C. hylemonae* DSM 15053 cultures. Some strains reduce *C. difficile* growth only when cultured with bile mixtures by converting PBAs into SBAs. Therefore, *C. hylemonae* DSM 15053 was inoculated into yBHI broth supplemented with 0.01% CA and CDCA, cultured for 24 h, and the SBA concentrations were quantified. Contrary to our expectations, DCA was rarely detected in *C. hylemonae* DSM 15053 (Fig. 3B). LCA was detected at levels comparable to *C. scindens* KCTC 5591, although interindividual variations were observed within the group. Meanwhile, PBAs and conjugated bile acids such as TDCA, TCA, UDCA, and TUDCA were relatively abundant in the *C. hylemonae* DSM 15053 group.

To assess metabolic changes induced by administration of *C. hylemonae* DSM 15053, we performed untargeted metabolomic analysis of stool samples. Distinct differences in metabolic profiles were identified between groups (Fig. 3C). Clusters from the *C. hylemonae* DSM 15053 group were positioned relatively left, although interindividual variations were observed. Nevertheless, this group clustered similarly to the *C. scindens* KCTC 5591 group, while being clearly separated from the PBS group, reflecting their metabolic differences. These results indicate that specific bacterial treatments induce significant metabolic changes in infected mice.

### Comparison of Gut Microbial Diversity

**Fig. 4:**
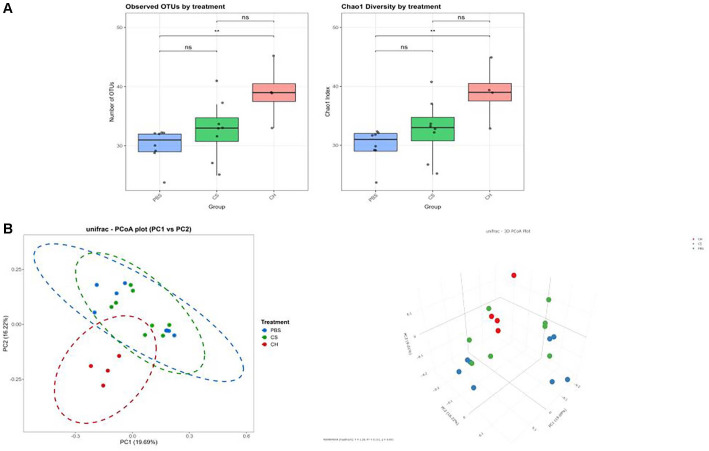
Comparison of microbial diversity. Alpha diversity (OTUs, Chao1) of analysis of microbiota in ceca of mice at 2 days after CDI (**A**). Statistical significance analyzed by the Kruskal–Wallis test with Dunn correction for multiple comparisons (n = 4 to 8 per group). The beta diversity through PCoA plot showing the difference in terms of OTU in ceca of mice at 2 days after CDI (**B**). (n = 4 to 8 per group), CS, *C. scindens*, CH, *C. hylemonae*.

Gut microbial diversity analysis was conducted to investigate differences in gut microbiota structure associated with CDI inhibition by *C. hylemonae* DSM 15053. Alpha diversity was analyzed using observed OTUs and Chao1 indices to measure species richness (Fig. 4A). In general, CDI is associated with a reduction in gut microbial diversity. In our study, administration of *C. hylemonae* DSM 15053 significantly increased the gut microbial diversity, as evidenced by higher alpha diversity indices in both observed OTUs (p = 0.0124) and Chao1 analyses (p = 0.0124). Also, Beta diversity was evaluated using PCoA, which revealed significant differences in microbial composition between groups. Notably, the *C. hylemonae* DSM 15053 group clustered separately from the PBS group, while the *C. scindens* KCTC 5591 group clustered more closely with the PBS group (Fig. 4B).

### Composition of Gut Microbiota and Relative Abundance of Dominant Bacterial Taxa at on 2 Days after the CDI

**Fig. 5:**
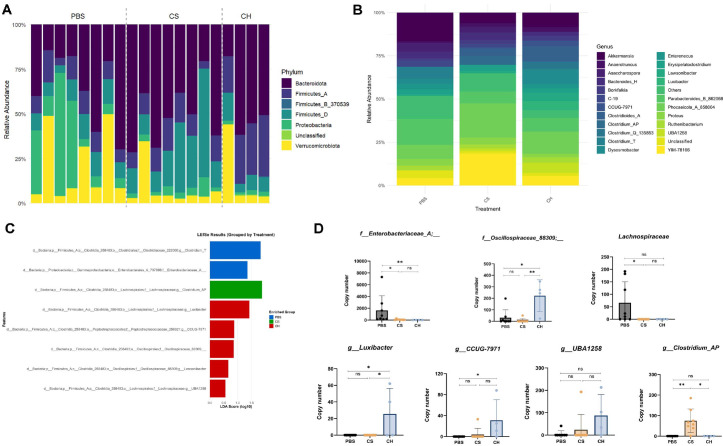
Composition of gut microbiota in the mice, Relative abundance of dominant bacterial taxa at on 2 days after the CDI. Relative abundances in fecal of all mice in phylum level (**A**) and genus level (**B**) were assessed. LEfSe analysis for all experimental groups (**C**) and relative abundance of genus (**D**) CS, *C. scindens*, CH, *C. hylemonae*.

The relative abundances of major bacterial OTUs in the ceca of mice administered *C. hylemonae* DSM 15053 at 5 days after CDI were identified. Our analysis shown that the microbiota of the ceca of mice are distinct according to the group. Proteobacteria was highly abundant group in cecal microbiota of PBS group, whereas observed scarcely in the *C. hylemonae* DSM 15053 group. On the other hands, Bacteroidota and Firmiticutes A, B were shown highly in the *C. hylemonae* DSM 15053 group, occupying less in PBS group (Fig. 5A and 5B). Next, we firstly proceeded LEfSe analysis to determine significant genus within experimental groups. As a result, among of 168 bacterial taxa, Enterobacteriaceae_A, Lachnospiraceae, Oscillospiraceae_88309, *g_Luxibacter*, *g_Clostridium*_AP were identified as primary bacterial taxa with high abundance in *C. hylemonae* DSM 15053 group compared to PBS (Fig. 5C and 5D).

### Spearman Correlation of *C. difficile* Copies and Microbial Community in *C. hylemonae* DSM 15053 Group

**Fig. 6:**
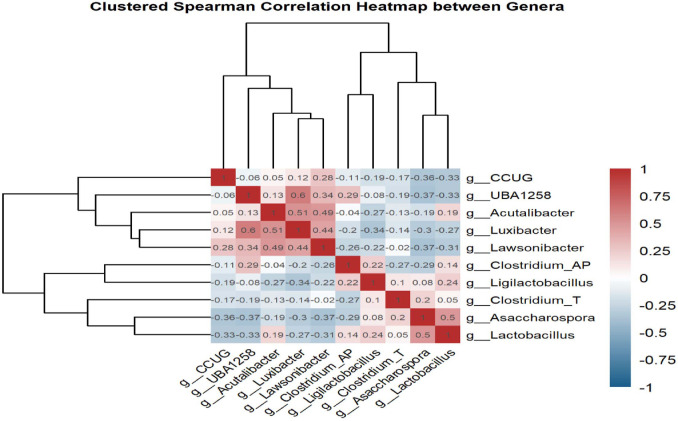
Spearman correlation of *C. difficile* copies and microbial community in *C. hylemonae* DSM 15053 group. Minimum cutoff was 0.5. Heatmap about correlation between significant genus.

Spearman correlation analysis was performed and visualized as a heatmap to better understand the CDI-inhibitory effects observed in the *C. hylemonae* DSM 15053 group (Fig. 6). In this group, key bacterial taxa exhibited overall positive correlations with each other. In contrast, in the PBS group, *Asaccharospora* and *Lactobacillus* were positively correlated with each other but negatively correlated with most of the significant taxa enriched in the *C. hylemonae* DSM 15053 group.

## Discussion

Antibiotic exposure is regarded as the predominant predisposing factor for CDI, as these agents disrupt the gut’s commensal microbial network that normally confers resistance to *C. difficile* [38, 39]. Accordingly, the present study sought to identify bacterial taxa capable of suppressing CDI and to uncover their underlying mechanisms through integrated analyses of microbiota composition and microbial metabolites.

The capacity of various *Clostridium* species to counteract CDI has been extensively described. For instance, *C. butyricum* MIYAIRI 588 suppresses *C. difficile* overgrowth by modulating both the gut microbial community and host metabolic pathways [40]. Likewise, *C. scindens* KCTC 5591—a bacterium capable of bile acid 7α-dehydroxylation—confers protection against CDI through mechanisms dependent on secondary bile acids [10]. In this study, we evaluated *C. hylemonae* DSM 15053, a strain harboring the *bai* operon, for its potential to mitigate CDI. *In vivo* administration of this strain alleviated weight loss, reduced mortality and clinical severity, and lowered *C. difficile* burden and toxin production, demonstrating marked inhibitory effects.

Recurrence remains a major challenge in CDI treatment, with 20–35% of patients relapsing after an initial episode and up to 60% experiencing subsequent recurrences [41, 42]. Complete clearance of *C. difficile* is therefore essential. Notably, *C. hylemonae* DSM 15053 significantly inhibited *C. difficile* growth both 1 and 8 days post-infection.

Loss of bile-metabolizing microbes and enzymes such as bile salt hydrolases and 7α-dehydroxylase promotes CDI by increasing PBAs that trigger spore germination and reducing SBAs that inhibit growth [43]. We therefore investigated SBAs levels (DCA and LCA). Unexpectedly, SBAs levels in the *C. hylemonae* group were lower than in the *C. scindens* KCTC 5591 group, consistent with previous reports. Despite harboring the baiCD operon, *C. hylemonae* is less efficient in converting PBAs to SBAs [11], likely due to missing genes such as baiA2, which encodes a short-chain dehydrogenase/reductase required for two steps of the 7α-dehydroxylation pathway [44]. Although baiA1 and potential redundancies may partially compensate, SBAs production was insufficient to fully explain its inhibitory effect.

Non-targeted metabolomics revealed distinct metabolic profiles between groups. The *C. hylemonae* cluster was close to that of *C. scindens* KCTC 5591 but separated from PBS, suggesting additional metabolites may contribute. Microbiome analysis further showed higher alpha and beta diversity in the *C. hylemonae* group. LEfSe analysis identified enrichment of taxa such as Lachnospiraceae, Enterobacteriaceae_A, *g_Luxibacter*, *g_Clostridium*_AP and Oscillospiraceae_88309 and others. Some of these, SCFA-producing Oscillospiraceae [45], and Lachnospiraceae consortia [46], have reported roles in CDI protection. These taxa may thus play key roles in the observed effect.

This study has limitations. Metabolomic differences between *C. scindens* KCTC 5591 and *C. hylemonae* DSM 15053 were modest, and the effective molecules and responsive host factors should be identified. In addition, In our study, we did not assess immune cell responses at all. Emerging evidence indicates that host immune responses - including T-cell, dendritic-cell, and innate immune pathways - interact with microbiota-mediated colonization resistance during CDI [47, 48]. Accordingly, future studies should investigate the immunological modulation induced by the administration of *C. hylemonae* DSM 15053 - such as enhanced mucosal barrier integrity, alterations in T-cell subsets, and changes in the cytokine milieu - which may represent another potential mechanism of protection against CDI.

**Fig. 7:**
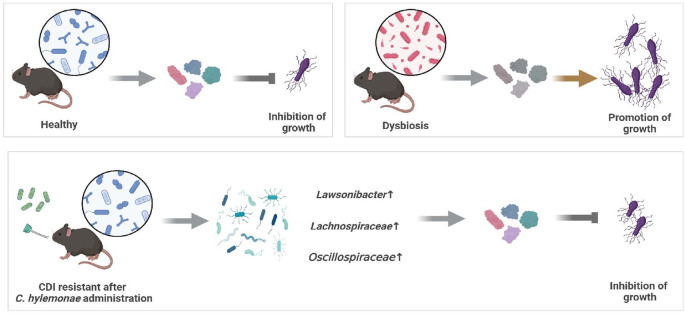
In a healthy gut, commensal microbiota confer colonization resistance by preventing the outgrowth and spore germination of *Clostridioides difficile*. However, indiscriminate antibiotic use disrupts the intestinal microbial ecosystem, leading to dysbiosis and subsequent expansion of *C. difficile*. Administration of *Clostridium hylemonae* DSM 15053 restores microbial diversity and suppresses *C. difficile* growth, thereby alleviating or preventing the development of *C. difficile* infection (CDI).

Nevertheless, this is the first report demonstrating a significant inhibitory effect of *C. hylemonae* DSM 15053 on CDI *in vivo*. While less efficient than *C. scindens* KCTC 5591 in SBAs production, *C. hylemonae* DSM 15053 appears to restore microbial diversity and richness, a key therapeutic goal in CDI. Current treatments such as FMT are limited by cost, donor availability, and variable outcomes, underscoring the need to identify specific protective strains (Fig. 7). In conclusion, the CDI-inhibitory effect of *C. hylemonae* DSM 15053 may stem primarily from restoring gut microbial diversity rather than SBAs production alone, warranting further validation in germ-free models.

## Conclusion

In summary, *C. hylemonae* DSM 15053 inhibited CDI in an animal model, as shown by reduced body weight loss, mortality, and improved clinical scores. Microbiome analysis revealed greater diversity and richness, with enrichment of taxa such as Lachnospiraceae, Enterobacteriaceae_A, and *g_Luxibacter*, *g_Clostridium*_AP and Oscillospiraceae_88309 and others. Metabolomic profiling identified distinct metabolite patterns, indicating that mechanisms beyond SBAs production may contribute to protection. Thus, the CDI inhibitory activity of *C. hylemonae* DSM 15053 may involve restoration of gut microbial diversity, and validation in germ-free models is warranted to clarify its therapeutic potential.

## Supplemental Materials

Supplementary data for this paper are available on-line only at http://jmb.or.kr.



## Figures and Tables

**Table 1:** Clinical scoring criteria for CDI symptoms.

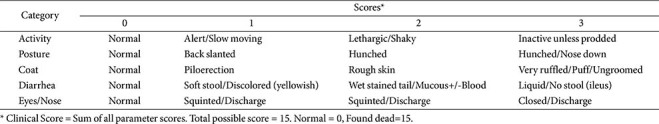

## References

[1] Czepiel J, Dróżdż M, Pituch H, Kuijper EJ, Perucki W, Mielimonka A (2019). *Clostridium difficile* infection: review. Eur. J. Clin. Microbiol. Infect. Dis..

[2] Koh E, Hwang IY, Lee HL, De Sotto R, Lee JWJ (2022). Engineering probiotics to inhibit *Clostridioides difficile* infection by dynamic regulation of intestinal metabolism. Nat. Commun..

[3] Bartlett JG (1979). Antibiotic-associated pseudomembranous colitis. Rev. Infect. Dis..

[4] Jeon SR (2024). The burden of *Clostridioides difficile* infection in Korea. J. Korean Med. Sci..

[5] Fujisaka S, Avila-Pacheco J, Soto M, Kostic A, Dreyfuss JM, Pan Hui (2018). Diet, genetics, and the gut microbiome drive dynamic changes in plasma metabolites. Cell Rep..

[6] Bien J, Palagani V, Bozko P (2013). The intestinal microbiota dysbiosis and *Clostridium difficile* infection: is there a relationship with inflammatory bowel disease. Ther. Adv. Gastroenterol..

[7] Leslie JL, Vendrov KC, Jenior ML, Young VB. 2019. The gut microbiota is associated with clearance of *Clostridium difficile* infection independent of adaptive immunity. *mSphere* **4:** e00187-19. https://doi.org/10.1128/mspheredirect.00698-18. 10.1128/mSphereDirect.00698-18 30700514 PMC6354811

[8] Buffie CG, Bucci V, Stein RR, McKenney PT, Ling L, Gobourne A (2015). Precision microbiome reconstitution restores bile acid mediated resistance to *Clostridium difficile*. Nature.

[9] Thanissery R, Winston JA, Theriot CM (2017). Inhibition of spore germination, growth, and toxin activity of clinically relevant *C. difficile* strains by gut microbiota-derived secondary bile acids. Anaerobe.

[10] Stellwag EJ, Hylemon PB (1978). Characterization of 7-α-dehydroxylase in *Clostridium leptum*. Am. J. Clin. Nutr..

[11] Kakiyama G, Pandak WM, Gillevet PM, Hylemon PB, Heuman DM, Kalyani Daita (2013). Modulation of the fecal bile acid profile by gut microbiota in cirrhosis. J. Hepatol..

[12] Yu JS. A rationally defined bacterial consortium restores colonization resistance against *Clostridioides difficile* through intestinal metabolites [dissertation]. Seoul: Seoul National University; 2023.

[13] Ridlon JM, Kang DJ, Hylemon PB (2010). Isolation and characterization of a bile acid inducible 7α-dehydroxylating operon in *Clostridium hylemonae* TN271. Anaerobe.

[14] García-Cañaveras JC, Donato MT, Castell JV, Lahoz A (2012). Targeted profiling of circulating and hepatic bile acids in human, mouse, and rat using a UPLC-MRM-MSvalidated method. J. Lipid Res..

[15] Putnam EE, Nock AM, Lawley TD, Shen A (2013). SpoIVA and SipL are *Clostridium difficile* spore morphogenetic proteins. J. Bacteriol..

[16] Li Y, Figler RA, Kolling G, Bracken TC, Rieger J, Stevenson RW (2012). Adenosine A2A receptor activation reduces recurrence and mortality from *Clostridium difficile* infection in mice following vancomycin treatment. BMC Infect. Dis..

[17] Sun X, Wang H, Zhang Y, Chen K, Davis B, H Feng (2011). Mouse relapse model of *Clostridium difficile* infection. Infect. Immun..

[18] Lyerly DM, Neville LM, Evans DT, Fill J, Allen S, Greene W (1998). Multicenter evaluation of the *Clostridium difficile* TOX A/B TEST. J. Clin. Microbiol..

[19] Hou M, Song P, Chen Y, Yang X, Chen P, Cao A (2024). Bile acids supplementation improves colonic mucosal barrier via alteration of bile acids metabolism and gut microbiota composition in goats with subacute ruminal acidosis. Ecotoxicol. Environ. Saf..

[20] Wells DA, Weil DA (2003). Directions in automated sample preparation of proteins. LCGC Europe.

[21] Dinsdale EA, Edwards RA, Bailey BA, Tuba I, Akhter S, McNair K (2013). Multivariate analysis of functional metagenomes. Front. Genet..

[22] Graham AS, Patel F, Little F, van der Kouwe A, Kaba M, Holmes MJ (2025). Using short-read 16S rRNA sequencing of multiple variable regions to generate high-quality results to a species level. Front. Bioinform..

[23] Bolyen E, Rideout JR, Dillon MR, Bokulich NA, Abnet CC, Al-Ghalith GA (2019). Reproducible, interactive, scalable and extensible microbiome data science using QIIME 2. Nat. Biotechnol..

[24] McDonald D, Jiang Y, Balaban M, Cantrell K, Zhu Q, Gonzalez A (2024). Greengenes2 unifies microbial data in a single reference tree. Nat. Biotechnol..

[25] Jeske JT, Gallert C (2022). Microbiome analysis via OTU and ASV-based pipelines-A comparative interpretation of ecological data in WWTP systems. Bioengineering.

[26] Glassman SI, Martiny JB. 2018. Broadscale ecological patterns are robust to use of exact sequence variants versus operational taxonomic units. *mSphere* **3:** e00148-18. https://doi.org/10.1128/msphere.00148-18. 10.1128/mSphere.00148-18 30021874 PMC6052340

[27] McMurdie PJ, Holmes S (2013). phyloseq: an R package for reproducible interactive analysis and graphics of microbiome census data. PLoS One.

[28] Wickham H. 2016. Data analysis. In: ggplot2: Elegant Graphics for Data Analysis. Springer, Cham. pp. 189-201. 10.1007/978-3-319-24277-4_9

[29] Oksanen J, Blanchet FG, Friendly M, Kindt R, Legendre P, *et al*. 2001. Vegan: community ecology package. R package version 2.0.

[30] Auguie B, Antonov A. 2017. Package ‘gridExtra’: miscellaneous functions for “grid” graphics. R package version 2.3.

[31] Wickham H. 2015. dplyr: A grammar of data manipulation. R package version 0.4.3.

[32] Wickham H, Francois R, Henry L, Muller K. 2023. dplyr: A grammar of data manipulation. R package version 1.1.4.

[33] Wickham H, Averick M, Bryan J, Chang W, McGowan LD, François R (2019). Welcome to the Tidyverse. J. Open Source Softw..

[34] Wickham H. 2016. Data analysis. In: ggplot2: Elegant Graphics for Data Analysis. Springer, Cham. pp. 189-201.

[35] Butts CT (2008). network: a package for managing relational data in R. J. Stat. Softw..

[36] Pedersen TL, Nissen JN, Eriksen PS, Albertsen M (2017). PanViz: interactive visualization of the structure of functionally annotated pangenomes. Bioinformatics.

[37] Cao Y, Dong Q, Wang D, Zhang P, Liu Y, Niu C (2022). microbiomeMarker: an R/Bioconductor package for microbiome marker identification and visualization. Bioinformatics.

[38] Douglas GM, Maffei VJ, Zaneveld JR, Yurgel SN, Brown JR, Taylor CM (2020). PICRUSt2 for prediction of metagenome functions. Nat. Biotechnol..

[39] Owens RC, Donskey CJ, Gaynes RP, Loo VG, Muto CA (2008). Antimicrobial-associated risk factors for *Clostridium difficile* infection. Clin. Infect. Dis..

[40] Kelly CP, Kyne L (2011). The host immune response to *Clostridium difficile*. J. Med. Microbiol..

[41] Hagihara M, Ariyoshi T, Kuroki Y, Eguchi S, Higashi S, Mori T (2021). *Clostridium butyricum* enhances colonization resistance against *Clostridioides difficile* by metabolic and immune modulation. Sci. Rep..

[42] Brandt LJ, Aroniadis OC, Mellow M, Kanatzar A, Kelly C, Park T (2012). Long-term follow-up of colonoscopic fecal microbiota transplant for recurrent *Clostridium difficile* infection. Am. J. Gastroenterol..

[43] Nelson WW, Scott TA, Boules M, Teigland C, Parente A, Unni S, Feuerstadt P (2021). Health care resource utilization and costs of recurrent *Clostridioides difficile* infection in the elderly: a real-world claims analysis. J. Manag. Care Spec. Pharm..

[44] Microbiology Spectrum (2023). Clostridium difficile infection by regulating bile acid metabolism. Microbiol. Spectr..

[45] Reed AD, Nethery MA, Stewart A, Barrangou R, Theriot CM (2020). Strain-dependent inhibition of *Clostridioides difficile* by commensal Clostridia carrying the bile acidinducible (bai) operon. J. Bacteriol..

[46] Tejada JN, Walters WA, Wang Y, Kordahi M, Chassaing B, Pickard J (2024). Prevention and cure of murine *C. difficile* infection by a Lachnospiraceae strain. Gut Microbes.

[47] Jarchum I, Liu M, Shi C, Equinda M, Pamer EG (2011). Toll-like receptor 5 stimulation protects mice from acute *Clostridium difficile* colitis. Infect. Immun..

[48] Leslie JL, Huang S, Opp JS, Nagy MS, Kobayashi M, Young VB (2015). Persistence and toxin production by *Clostridium difficile* within human intestinal organoids result in disruption of epithelial paracellular barrier function. Infect. Immun..

