# Corrigendum to: Attenuation of *Clostridioides difficile* Infection by *Clostridium hylemonae*

**DOI:** 10.4014/jmb.2026.3603.C03

**Published:** 2026-06-10

**Authors:** Sueun Choi, Heewon Kwon, Woon-Ki Kim, GwangPyo Ko

**Affiliations:** 1 Department of Environmental Health Sciences, Graduate School of Public Health, Seoul National University, Seoul 08826, Republic of Korea; 2 Brain Korea for Global Leader of Better Environmental health (BK4GLOBE), Republic of Korea; 3 Institute of Health and Environment, Seoul National University, Seoul 08826, Republic of Korea

In the article titled “Attenuation of *Clostridioides difficile* Infection by

*Clostridium hylemonae*,” we would like to request corrections to the author information.

First, the English name of the co-first author was incorrectly published as **Heewon Kwon**. The correct spelling is **Heeun Kwon**, and we kindly request that this be updated accordingly.

Second, we would like to request the addition of the co-first author designation symbol (†) to both **Sueun Choi** and **Heeun Kwon** to indicate that they contributed equally to this work as co-first authors.

The corrected author list is as follows, reflecting the correction of the author name from **Heewon Kwon** to **Heeun Kwon** and the addition of the co-first author designation (†).

Sueun Choi^†1^, Heeun Kwon^†1^, Woon-Ki Kim^1,2^, and GwangPyo Ko^1,3*^

^†^These authors contributed equally to this work.

We acknowledge these corrections and confirm that they do not affect the results, interpretations, or conclusions of the article.

The authors would like to apologies for any inconvenience caused. (http://jmb.or.kr)

