# Supplementary figures and images for: Attenuation of Clostridioides difficile Infection by Clostridium hylemonae
Source: J Microbiol Biotechnol. 2026 Jan 13;36:e2510017. doi: 10.4014/jmb.2510.10017 (PMC12828130; doi:10.4014/jmb.2510.10017)

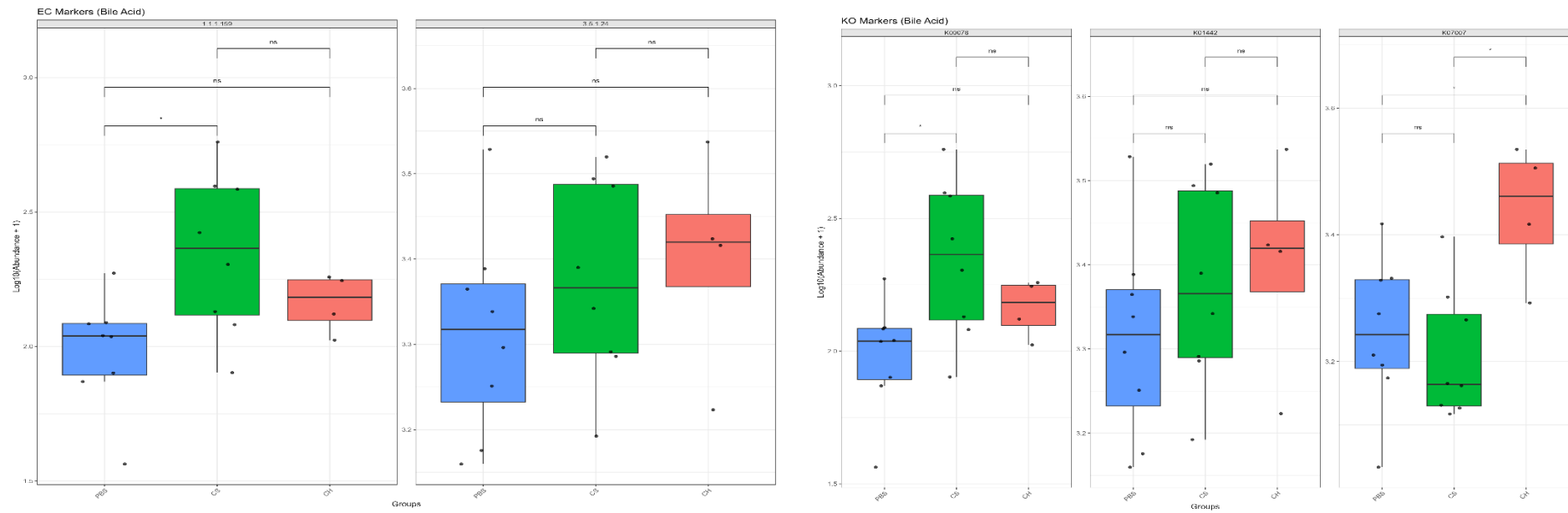

Figure S1.

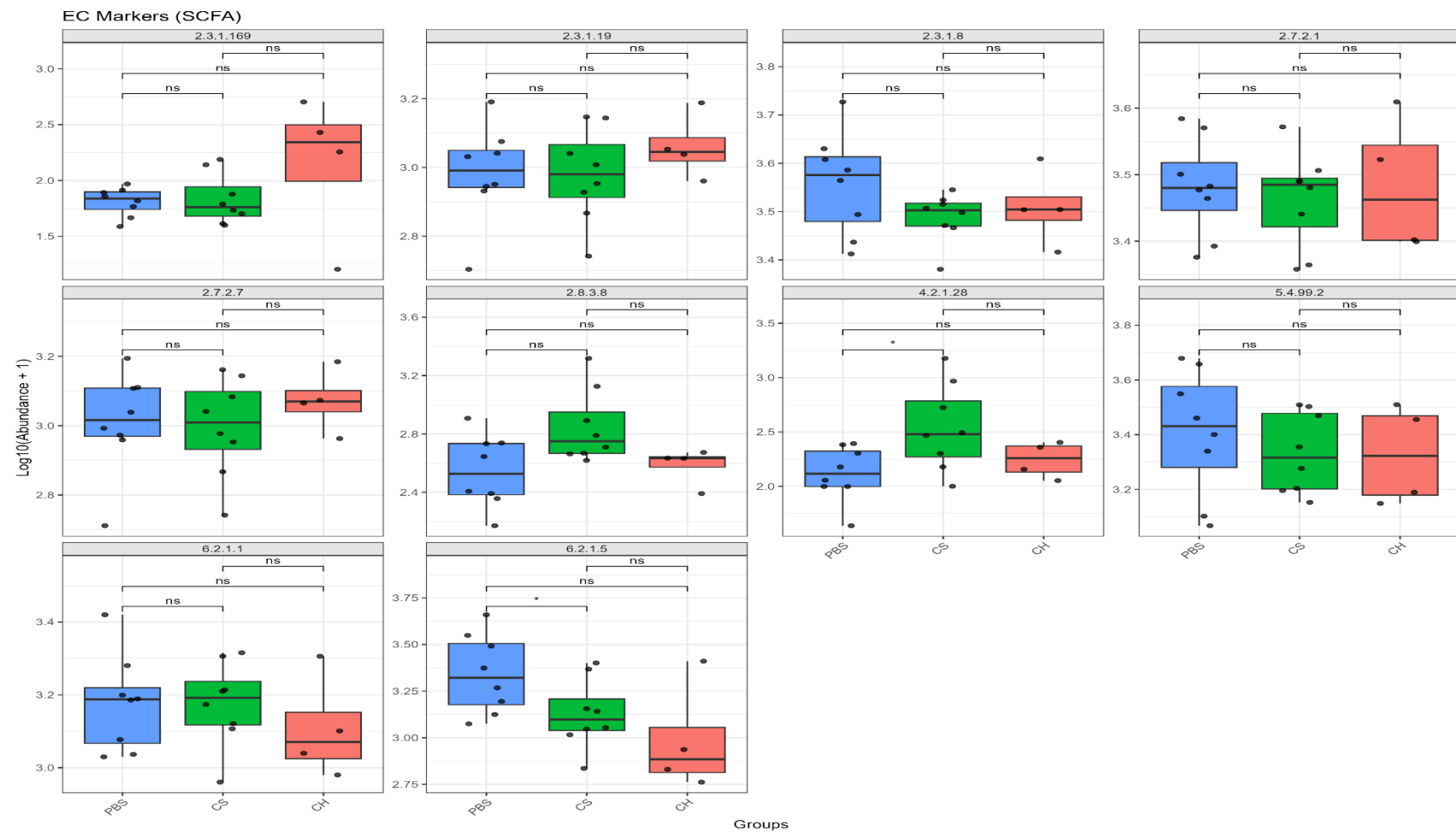

Figure S2.

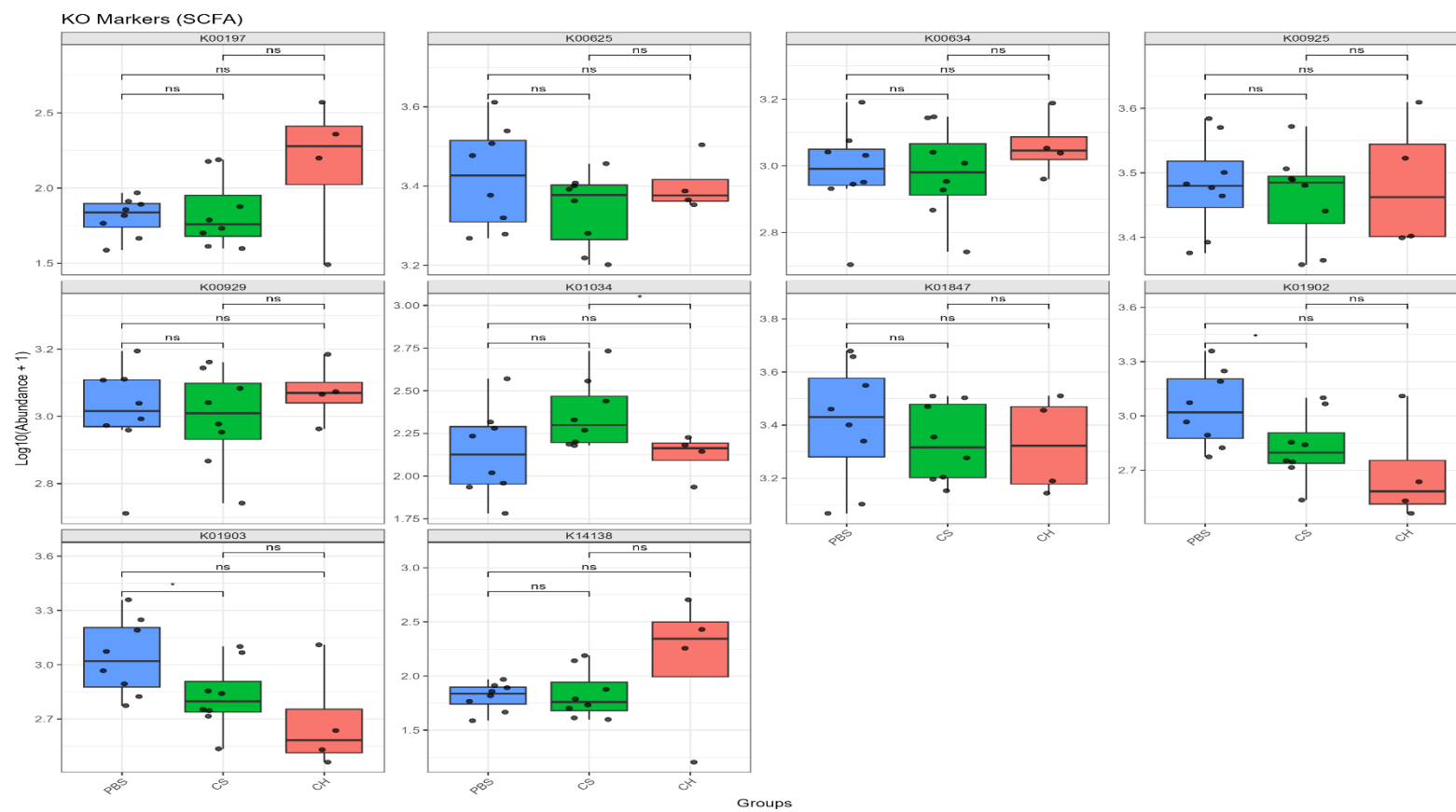

Figure S3.

Supplement: Supplementary file 1 [file jmb-36-e2510017-supple.pdf]
